# Developing Country-Specific Charlson Comorbidity Index Mappings for Use With German Administrative Data: Methodological Comparative Study

**DOI:** 10.2196/93923

**Published:** 2026-09-03

**Authors:** Piotr Paweł Sokołowski, Michael Hagmann, Máté E Maros, Gaetan Kamdje Wabo, Josefine Maria Meerjanssen, Fabian Siegel

**Affiliations:** 1 Department of Biomedical Informatics Mannheim Institute for Intelligent Systems in Medicine (MIISM), Medical Faculty Mannheim Heidelberg University Mannheim, Baden-Wuerttemberg Germany; 2 Department of Medical Statistics, Biomathematics and Information Processing Medical Faculty Mannheim Heidelberg University Mannheim, Baden-Wuerttemberg Germany

**Keywords:** Charlson Comorbidity Index, *International Classification of Diseases, 10th Revision, German Modification*, *ICD-10-GM*, real-world data, comorbidity mapping, risk adjustment, electronic health records, Germany, automated scoring, Quan mapping, Deyo adaptation

## Abstract

**Background:**

The Charlson Comorbidity Index (CCI) is widely used to quantify comorbidity burden in observational research. Applying it to real-world data requires accurate *International Classification of Diseases*–based mappings. The commonly used mapping does not reflect current German coding standards.

**Objective:**

This study aimed to develop year-specific mappings (2004 to 2026) based on the German modification of the *International Classification of Diseases, 10th Revision* (*ICD-10-GM*), for the CCI with an open-source implementation and compare them with the commonly used mapping.

**Methods:**

For each year, mappings were curated via independent dual review with consensus. In 515,827 inpatient cases from a German tertiary center (2010-2024), year-appropriate mappings and the commonly used mapping were applied, and the results were pooled into 2 corresponding datasets that were subsequently compared. Agreement was assessed using the intraclass correlation coefficient (2-way mixed-effects model for absolute agreement based on single measurement), and the significance of the discordances was measured using the McNemar test.

**Results:**

Agreement was very high; 3.1% (16,136/515,827) of cases differed. A single code was present in 48.9% (7896/16,136) of the discordant cases and was intentionally excluded in the new mappings as it was not diagnostic for the category it was assigned to. Discordant results were found in 58.8% (10/17) of the categories (“peripheral vascular disease,” “mild liver disease,” and “severe liver disease”) being present in, respectively, 51.7% (8339/16,136), 17.4% (2813/16,136), and 16.4% (2657/16,136) of the discordant cases. After filtering the dataset to only include observations with any diagnosis of a malignant disease, 6.9% (9377/136,607) of cases differed, and 79.3% (7434/9377) of those differences were caused by the “peripheral vascular disease” category, suggesting significant discrepancies for specific research questions.

**Conclusions:**

We provide transparent, year-specific *ICD-10-GM* (2004-2026) mappings and an open-source tool for CCI calculation. The mappings align with current German coding practice and enable reproducible, year-appropriate comorbidity adjustment using administrative data.

## Introduction

### Background

In recent years, the increasing availability of real-world data from electronic health records, insurance claims, and clinical registries has opened new avenues for large-scale observational research [1]. These data sources often comprise hundreds of thousands of patient records and present opportunities to explore treatment effectiveness, patient outcomes, and health care use beyond the constraints of randomized controlled trials [2]. However, the heterogeneity and complexity of such datasets also necessitate robust methods for patient characterization, automated grouping, and risk stratification.

The Charlson Comorbidity Index (CCI) is one of the most widely used tools for quantifying a patient’s burden of comorbid diseases. Initially developed to predict 1-year mortality based on the presence of specific comorbid conditions [3], the CCI has since become a widely used metric in retrospective cohort studies, serving as a predictor of morbidity, mortality, and health care outcomes across a broad range of clinical domains [4]. In the clinical setting, its use is suggested in several clinical guidelines, for example, the 2025 European Association of Urology guideline [5]; the German S3 guidelines on prostate cancer [6], multiple myeloma [7], and diverticulitis [8]; and the National Comprehensive Cancer Network’s comprehensive geriatric assessment [9].

Over the years, many different adaptations of the original CCI have been created by adding or removing categories or changing their weights. These versions have been validated in different ways; however, they have rarely outperformed the original [4].

In 1992, Deyo et al [10] adapted the CCI for use with *International Classification of Diseases* (*ICD*)*, Ninth Revision, Clinical Modification* (*ICD-9-CM*), administrative databases and modified it slightly, merging malignancy categories (solid tumors, lymphoma, and leukemia) into one, resulting in 17 instead of 19 different categories.

From that point on, a blueprint existed for using extracted administrative data to calculate a comorbidity index. Later, Sundararajan et al [11] translated the *ICD-9-CM* codes into the *ICD, 10th Revision* (*ICD-10*)*, Australian Modification*. A year later, Quan et al [12] revised the codes again, creating a new *ICD-10* code list for every comorbidity.

Quan et al [12] intended to keep their mapping country independent by restricting the *ICD* codes to 4 characters (ie, the first letter and 3 digits) [12]. However, the list of *ICD* codes has since undergone substantial revisions, introducing new subcodes and modifying disease definitions, with many countries developing their own versions or modifications, such as the *ICD-10, German Modification* (*ICD-10-GM*). These refinements have been made predominantly at the most granular level (the fourth digit or the fifth character of a code), making country-specific mappings necessary—at least theoretically.

Despite these changes, the mapping published by Quan et al [12] in 2005 remains one of the most popular approaches and continues to be frequently cited in today’s research (approximately 10,000 citations on Web of Science as of January 29, 2026) while also acting as the basis of automated CCI calculations in popular Python or R packages [13,14]. To our knowledge, there is currently no openly available mapping that has been developed specifically for German administrative data. Therefore, the aim of our study was to develop an open-source tool enabling reproducible comorbidity adjustment for German real-world data. We benchmarked our approach against the mappings by Quan et al [12] in a large cohort of 515,827 inpatient cases from a German tertiary care center (2010-2024) using year-specific mappings for each case and demonstrated that our mappings are better aligned with current German coding practice.

### Objectives

To address this potential gap between the current German coding practices and the available mapping, we developed new mappings for calculating the CCI using the *ICD-10-GM* for the years 2004 to 2026. The goal of this study was to (1) create updated, country-specific, year-specific CCI mappings ensuring compatibility with current German coding standards; (2) compare the newly developed mappings with the widely available mapping by Quan et al [12] from 2005 evaluating agreement and category-level differences, thus investigating whether the new mapping is the optimal way to achieve the above-mentioned compatibility; and (3) provide an easy-to-use, transparent, open-source tool using the mapping, thus facilitating the optimal comorbidity classification for German patient data and future adaptations.

We aimed to improve the compliance of the automated comorbidity classification and scoring with actual clinical practice in German hospitals. This will enable more adequate stratification of the patients in retrospective clinical research.

As the goal of this project was not the revalidation of clinimetric properties of the CCI but, rather, updating the currently used mapping and keeping it compatible with other country-specific CCI adaptations, the very same categories and weights were used as in the version by Quan et al [12].

## Methods

### Mappings

As a reference, we used the *ICD-10* mapping as published by Quan et al [12] in 2005. We then constructed a single new mapping tailored to the 2024 *ICD-10-GM* using a prespecified, multistep process as follows.

First, we adopted the adaptation by Deyo et al [10] of the CCI (17 categories instead of 19), effectively setting the same framework for our mapping as Quan et al [12] did in 2005.

Second, 2 clinicians (FS and PPS; FS also being a coding expert with a decade of experience) independently screened the 2024 *ICD-10-GM* list as provided online by the German Federal Institute for Drugs and Medical Devices (Bundesinstitut für Arzneimittel und Medizinprodukte; BfArM) to identify candidate codes for every category. The selection process was based first on the clinical term defining the category and on the descriptions of the comorbid categories from the 1987 paper by Charlson et al [3].

Third, the independently created code sets were compared. Discrepancies were enumerated and resolved in a consensus meeting. For each discrepant code, discussion was held until a consensus was achieved. In cases in which no consensus was achieved, a disease-specific coding expert was consulted.

The 2024 iteration was developed first because it corresponded to the most recent year available in our patient data.

After establishing the 2024 mapping, we audited each *ICD-10-GM* annual release (2004-2026) and compiled a year-specific list of added, removed, split, merged, or redefined codes for each year. These changes were then independently revised by the same 2 clinicians, and the proposed changes in the mappings for each year underwent the same multistep process explained above. The resulting mappings were intended to be applied according to each case’s discharge year.

A new feature as compared to the mapping by Quan et al [12] was the introduction of Boolean rules in some of the categories, which enabled more specific scoring (eg, code A and code B score points when they are both present but not separately).

To summarize, the new mappings included new sets of *ICD* codes chosen according to the above-mentioned algorithm. For each year, a new mapping was created, which enabled coding up to the most granular level, unlike the mapping by Quan et al [12], where the limitation of 4 characters per code was introduced with the intention of the single mapping being transferable to subsequent years and other countries’ coding practices. The next level of granularity was achieved by the introduction of Boolean rules so that some codes could only score points in specific combinations according to German coding guidelines.

### Algorithm Design

To practically use the mapping and calculate the CCI, we created a Python library with one core function requiring one or more *ICD* codes and a mapping definition as input and returning the score (number of points) and the scored categories.

The mapping definition was intentionally separated from the CCI scoring logic to enable easy modifications without having to change the central component of the algorithm.

The library consists of 24 separate mappings: a mapping described and validated beforehand by Quan et al [12] in an unchanged form and the 23 mappings built by authors FS and PPS based on the *ICD-10-GM* for years 2004 to 2026.

The algorithm was then applied to a large dataset of German patients’ administrative data.

The results obtained by applying our year-specific mappings—for each case, the mapping for the corresponding year was applied—were pooled and compared with the results obtained using the mapping by Quan et al [12], which stayed the same throughout all years.

The mappings were developed—as mentioned above—for years 2004 to 2026. However, as our study population included records from the years 2010 to 2024 (see the Study Population and Data Source section), only the mappings for those years were deployed.

### Statistical Analyses

The statistical analyses were conducted in Python (version 3.12.3; Python Software Foundation) [15] and R (version 4.4.2; R Foundation for Statistical Computing) [16]. For each case, we computed 2 total CCI scores: one using the year-specific *ICD-10-GM* mapping (selected by discharge year) and one using the mapping by Quan et al [12].

We first calculated the proportion of cases with different total CCI scores between the 2 approaches. To quantify the agreement, the intraclass correlation coefficient (ICC) was calculated. We used a 2-way mixed-effects model for absolute agreement based on single measurement because we wanted to assess only the absolute agreement of the scores (and not their consistency) between the 2 (fixed) alternative mappings [17]. The dependent variables in this analysis were the CCI scores per case. The analysis was conducted using the *irr* package [18] in R. We reported the resulting ICC and *P* value.

For each of the 17 categories, we applied the McNemar test to the discordant pairs. When the number of discordant pairs was less than 25, we used the exact binomial test; otherwise, continuity-corrected chi-square approximation was used. Tests were 2 sided, with an α of .05. Given the explorative nature of the analysis, no adjustment for multiplicity was applied. The calculation of the McNemar test was performed using the *statsmodels* library (version 0.15.0) [19] in Python.

Category-level concordance was displayed as a diverging bar chart ordered by total discordant count. Figures were produced using the *matplotlib* package (version 3.5.3) [20] in Python. Further investigation was undertaken when a significant difference was exhibited to determine whether the difference was the result of erroneous or better representation of *ICD* codes by one of the approaches. For the category with most total discrepancies, an additional bar chart was created to show its proportion in different diverging scores.

To assess the score-level impact of mapping discordance, we calculated ΔCCI for each case as the difference between the CCI obtained by our mapping and by the mapping by Quan et al [12]. We plotted the distribution of nonzero ΔCCI values.

### Study Population and Data Source

#### Overview

Our dataset comprised all inpatient cases treated at the University Medical Center Mannheim in the years 2010 to 2024. The data were pseudonymized before the analysis and included key administrative and clinical variables: a unique patient identifier, a case ID, the hospital admission time stamp, age, and a list of *ICD*-coded diagnoses per case. These data formed the basis for validating and testing the comorbidity scoring logic under real-world conditions.

The University Medical Center Mannheim is a tertiary care hospital and academic teaching hospital affiliated with Heidelberg University. As a maximum care provider, it covers nearly all medical specialties and treats a broad and diverse patient population, including highly complex and multimorbid cases. Its data structure and long-term digital archiving make it an ideal real-world reference for developing tools aimed at retrospective secondary use of routine clinical data.

#### Study Population Characteristics

**Figure 1:**
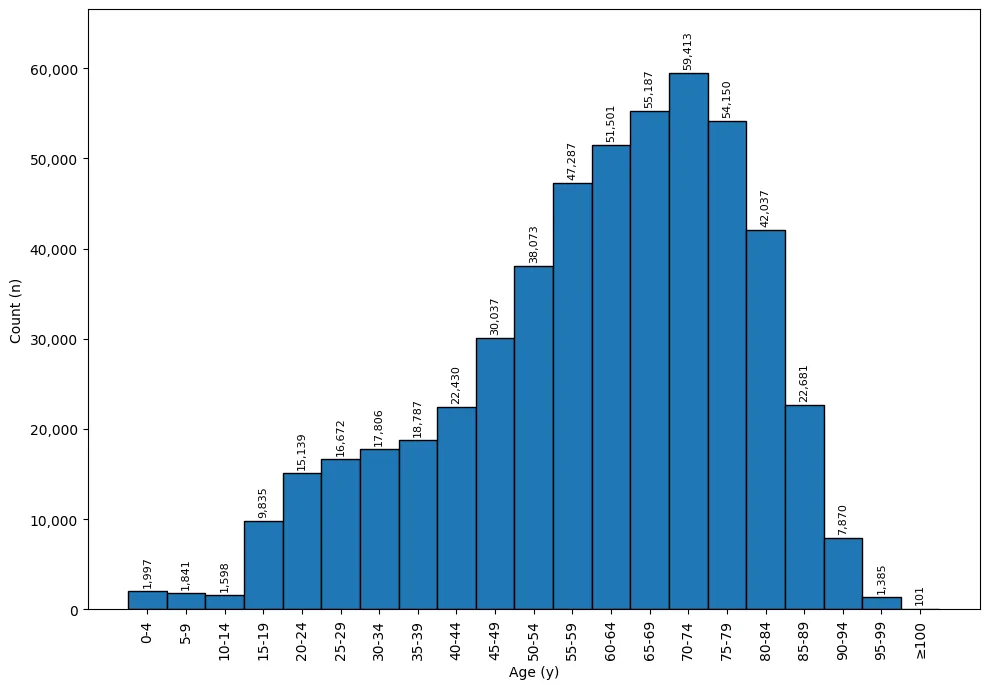
Age distribution in the study cohort.

A total of 515,827 cases were processed. As the data were pseudonymized for privacy protection reasons, age in the form of an integer was the only demographic information available. The age distribution of the study cohort is shown in Figure 1. Patient age ranged from 0 to 124 years, with a mean age of 59.31 (SD 18.71) years.

### ICD Coding System

The code list with descriptions (*ICD-10-GM* 2004-2026) was obtained from the official website of the BfArM [21].

### Ethical Considerations

The study was approved by the Ethics Committee II, Medical Faculty Mannheim, Heidelberg University (approval ID 2024-885). The requirement for informed consent was waived due to the retrospective analysis of pseudonymized data.

## Results

### Mappings

Table 1 shows a comparison of the mapping by Quan et al [12] from 2005 and our mapping based on the *ICD-10-GM* from 2024 (developed using the process detailed in the Methods section). For clarity purposes, the other year-specific mappings are not presented in this table but are openly available online (see the Data Availability section).

**Table 1:** Comparison of *International Classification of Diseases (ICD), 10th Revision (ICD‑10)*, code mappings for the Charlson Comorbidity Index (CCI)^a^.

	The authors’ *ICD-10-GM*^b^ 2024 mapping	Original mapping by Quan et al [12]
Liver disease, moderate to severe	I98.2 AND (K70.x^c^ OR K71.x OR K74.x), I98.3 AND (K70.x OR K71.x OR K74.x), K70.4x, K71.1, K72.7x, K72.x, K74.71, K74.72, and K76.5-K76.7	I85.0, I85.9, I86.4, I98.2, K70.4, K71.1, K72.1, K72.9, K76.5, K76.6, and K76.7
Liver disease, mild	B18.x, K70.x, K71.x (except for K71.1), K73.x, K74.x (except for K74.71 and K74.72), K76.x (except for K76.5-K76.7), K77.x, and Z94.4	B18.x, K70.0-K70.3, K70.9, K71.3-K71.5, K71.7, K73.x, K74.x, K76.0, K76.2-K76.4, K76.8, K76.9, and Z94.4
Malignancy, not metastatic	C00.x-C26.x, C30.x-C34.x, C37.x-C41.x, C43.x, C45.x-C58.x, C60.x-C76.x, C81.x-C86.x, C88.x, and C90.x-C97.x	C00.x-C26.x, C30.x-C34.x, C37.x-C41.x, C43.x, C45.x-C58.x, C60.x-C76.x, C81.x-C85.x, C88.x, and C90.x-C97.x
Malignancy, metastatic	C77.x-C80.x	C77.x-C80.x
AIDS	B20-B22, B24, and U60.3	B20.x-B22.x and B24.x
Cerebrovascular	G45.x, G46.x, H34.0, and I60.x-I69.x	G45.x, G46.x, H34.0, and I60.x-I69.x
Chronic pulmonary disease	I27.8, I27.9, J40.x-J47.x, J60.x-J67.x, J68.4, J70.1, J70.3, J84.1x, J96.1, J99.2x, and T86.81	I27.8, I27.9, J40.x-J47.x, J60.x-J67.x, J68.4, J70.1, and J70.3
Rheumatic disease	M05.x, M06.x, M31.5, M32.x-M34.x, M35.1, M35.3, and M36.0	M05.x, M06.x, M31.5, M32.x-M34.x, M35.1, M35.3, and M36.0
Dementia	F00.x-F03.x, F05.1, G30.x, G31.0, G31.1, and U63.x	F00.x-F03.x, F05.1, G30.x, and G31.1
Diabetes mellitus with chronic complications	E10.2-E10.5, E10.7, E11.2-E11.5, E11.7, E12.2-E12.5, E12.7, E13.2-E13.5, E13.7, E14.2-E14.5, and E14.7	E10.2-E10.5, E10.7, E11.2-E11.5, E11.7, E12.2-E12.5, E12.7, E13.2-E13.5, E13.7, E14.2-E14.5, and E14.7
Diabetes mellitus without chronic complications	E10.0, E10.1, E10.6, E10.8, E10.9, E11.0, E11.1, E11.6, E11.8, E11.9, E12.0, E12.1, E12.6, E12.8, E12.9, E13.0, E13.1, E13.6, E13.8, E13.9, E14.0, E14.1, E14.6, E14.8, and E14.9	E10.0, E10.1, E10.6, E10.8, E10.9, E11.0, E11.1, E11.6, E11.8, E11.9, E12.0, E12.1, E12.6, E12.8, E12.9, E13.0, E13.1, E13.6, E13.8, E13.9, E14.0, E14.1, E14.6, E14.8, and E14.9
Heart failure	I09.9, I11.0x, I13.0, I13.2, I25.5, I42.x, I43.x, I50.x, and P29.0	I09.9, I11.0, I13.0, I13.2, I25.5, I42.0, I42.5-I42.9, I43.x, I50.x, and P29.0
Kidney disease	I12.0x, I13.1, I13.2, N03.2-N03.7, N05.2-N05.7, N18.x, N19, N25.0, Z49.1, Z49.2, Z94.0, and Z99.2	I12.0, I13.1, N03.2-N03.7, N05.2-N05.7, N18.x, N19.x, N25.0, Z49.0-Z49.2, Z94.0, and Z99.2
Myocardial infarction	I21.x, I22.x, and I25.2x	I21.x, I22.x, and I25.2
Paraplegia and hemiplegia	G04.1, G11.4, G80.0-G80.2, G81.x, G82.x, G83.0, G83.5, and G83.9	G04.1, G11.4, G80.1, G80.2, G81.x, G82.x, G83.0-G83.4, and G83.9
Peptic ulcer	K25.x-K28.x	K25.x-K28.x
Peripheral vascular disease	I70.x, I71.x, I73.1, I73.8, I77.1, I79.0, I79.2, K55.1, K55.9, Z95.88, and Z95.9	I70.x, I71.x, I73.1, I73.8, I73.9, I77.1, I79.0, I79.2, K55.1, K55.8, K55.9, Z95.8, and Z95.9

^a^Shown are the newly developed ICD‑10, German Modification, 2024 mapping and the original ICD‑10 mapping by Quan et al [12]. The table lists ICD codes and logical conditions for each of the 17 CCI categories.

^b^ICD-10-GM: International Classification of Diseases, 10th Revision, German Modification (2024 edition).

^c^“.x” denotes the inclusion of all subcodes.

### Algorithm

#### Overview

The source code of the project is publicly available under the MIT License (see the Data Availability section). It is openly maintained and versioned to support transparency, reproducibility, and community collaboration.

The core programmatic interface of the library is the function “calculate_score(icd_codes, mapping, exact_codes)”:

- “icd_codes”—a single *ICD-10* code or a list of codes
- “mapping”—selected mapping variant
- “exact_codes”—if “True,” requires exact code matching in order to score points in a given category; “False” (default) applies prefix-based matching so all matching subcodes score points

The function returns a tuple (score and categories), where “score” is an integer from 0 to 29 and “categories” constitutes a list of strings representing the comorbidity groups that obtained positive scores. Mappings with weights and logical operators are stored in a structured JSON file (Textbox 1).

**Example function call.**

**Input**

calculate_score(icd_codes=['E10.0', 'K71.1'], mapping = 'icd10gm_2024', exact_codes = False)

**Output**

(3, ['dm_simple', 'liver_severe'])

#### Exploration of Mapping Concordance

The first step of data exploration was the direct comparison of the discordant cases in terms of conflicting CCI scores. The “exact_codes” parameter of the algorithm was set to “False.”

The proportion of records that produced different results when comparing the 2 mappings was 3.1% (16,136/515,827). After filtering the cohort to only contain cases with any malignancy diagnosis (to investigate the effects in a specialized cohort), this proportion increased to 6.9% (9377/136,607).

#### Agreement Between Mappings and Category-Level Discordance

To quantify overall agreement between the updated mapping and the original mapping by Quan et al [12], we calculated the ICC for the resulting scores. The ICC was 0.992 (*P*<.001), indicating excellent agreement between the comorbidity scores produced by the 2 mappings.

Despite this high overall concordance, category-level discordances were observed. Table 2 shows the number of cases that were scored in each comorbidity category using our mapping, the number of discordant cases, and their percentage.

**Table 2:** Category-level discordances. Categories are sorted by number of discordant cases (first column) in descending order.

	Discordant, n^a^	Discordant—only positive under Quan et al [12] mapping, n^b^	Discordant—only positive under our mapping, n^c^	Positive under both mappings, n	Disagreement, (%^d^)
Peripheral vascular disease	8339	8339	0	20,837	28.58
Liver disease, mild	2813	1092	1721	11,352	19.86
Liver disease, severe	2657	849	1808	3876	40.67
Hemiplegia or paraplegia	1719	1636	83	16,284	9.55
Chronic pulmonary disease	1160	0	1160	28,707	3.88
Heart failure	1034	0	1034	41,040	2.46
Dementia	171	0	171	12,134	1.39
Malignancy (nonmetastatic)	99	0	99	98,465	0.10
Renal disease	32	13	19	32,709	0.10
AIDS	20	0	20	778	2.51
Metastatic malignancy	0	0	0	38,043	0
Myocardial infarction	0	0	0	22,252	0
Cerebrovascular disease	0	0	0	29,956	0
Diabetes, with complications	0	0	0	14,897	0
Peptic ulcer	0	0	0	4188	0
Diabetes, uncomplicated	0	0	0	54,128	0
Rheumatologic disease	0	0	0	3731	0

^a^The number of cases positive in only 1 of the 2 mappings.

^b^The number of cases positive for the category under the mapping by Quan et al [12] but negative under ours.

^c^The number of cases positive for the category under our mapping but negative under the mapping by Quan et al [12].

^d^Disagreement among cases positive under either mapping, calculated using the following formula: (discordant—only positive under Quan et al [12] mapping + discordant—only positive under our mapping)/(discordant—only positive under Quan et al [12] mapping + discordant—only positive under our mapping + positive under both mappings) × 100%.

**Figure 2:**
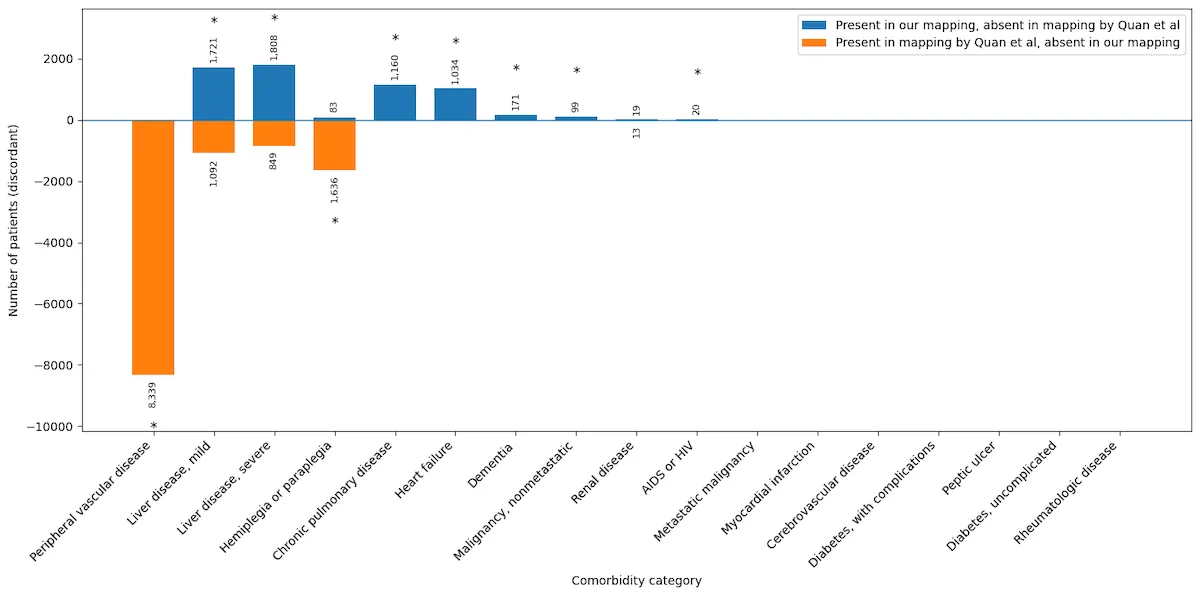
Discordant cases by comorbidity category. Categories are ordered left to right by total discordant count. Upward bars indicate cases identified by our mapping only; downward bars indicate cases identified by the mapping by Quan et al [12] only. *P<.05 (2-sided McNemar test; category-level 2-sided, unadjusted *P*<.001 for peripheral vascular disease; chronic pulmonary disease; heart failure; hemiplegia or paraplegia; liver disease, severe; dementia; liver disease, mild; malignancy [nonmetastatic]; and AIDS or HIV). *P*=.38 for renal disease (not significant). In the rest of the categories, there were no discordances.

**Figure 3:**
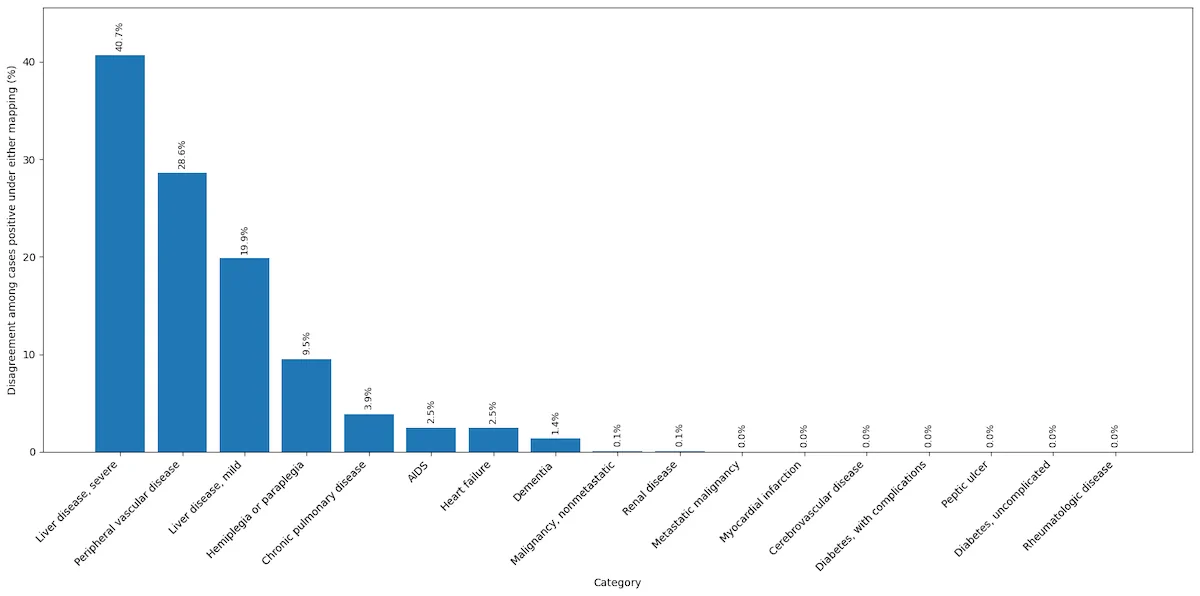
Percentage disagreement among cases positive for either mapping by comorbidity category. The bar plot shows the proportion of cases in which only 1 of the 2 mappings identified a given comorbidity among all cases identified as positive by at least one mapping.

Figure 2 shows the discordant case counts per comorbidity category, and Figure 3 shows the disagreement among cases who were positive for either mapping for each comorbidity category in percentages according to the following formula: (discordant—only positive under the Quan et al [12] mapping + discordant—only positive under our mapping)/(discordant—only positive under the Quan et al [12] mapping + discordant—only positive under our mapping + positive under both mappings) × 100%. Categories are ordered in descending order, so those on the left drive the most differences.

The largest discrepancy between the mapping by Quan et al [12] and ours was observed for “peripheral vascular disease.” This category was scored positively using the mapping by Quan et al [12] and negatively using our mapping for 51.7% (8339/16,136) of all discordant results. It was followed by the categories “mild liver disease,” and “severe liver disease” in, respectively, 17.4% (2813/16,136), and 16.4% (2657/16,136) of all discordant results. After filtering the data to only include the observations with a malignant disease diagnosis, this discordance in the category “peripheral vascular disease” affected 79.3% (7434/9377) of all the discordant cases.

**Figure 4:**
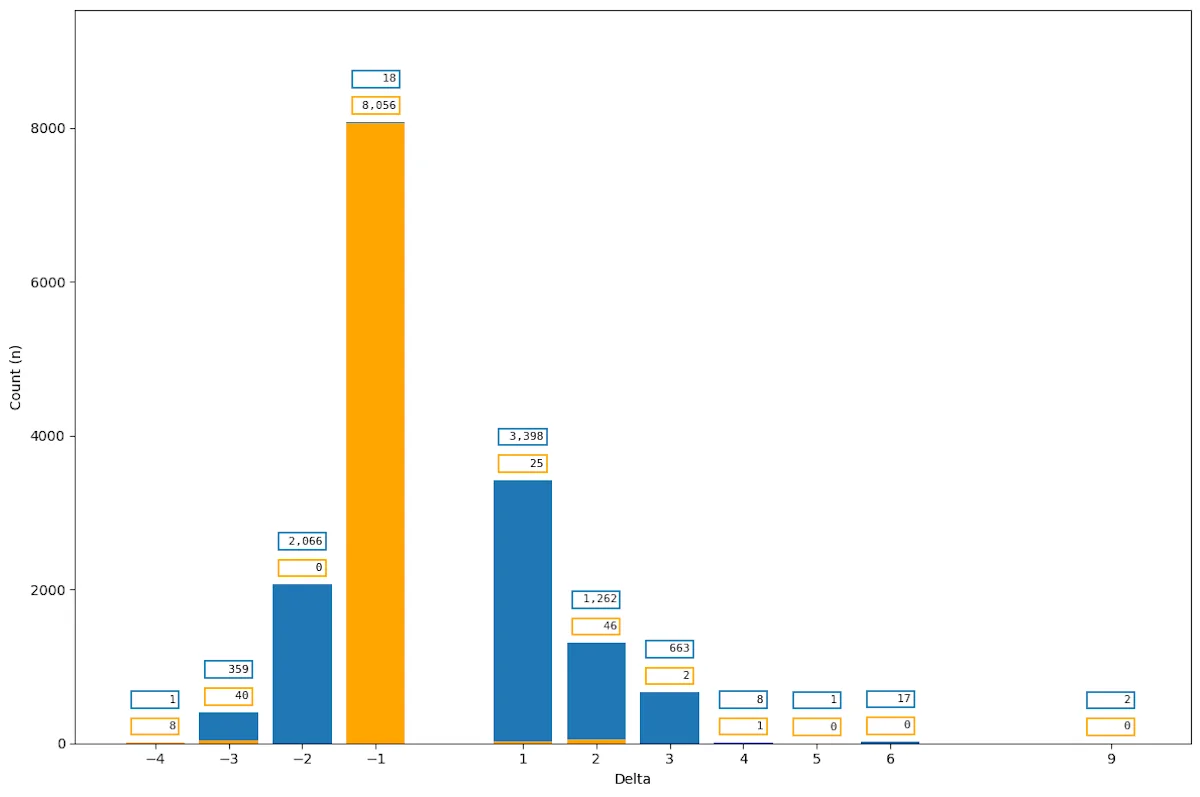
Distribution of score differences between the 2 mappings. Delta represents the score difference between our mapping and the mapping by Quan et al [12], calculated as score assigned by our mappings minus the score assigned by the mapping by Quan et al [12]. Bars show the number of cases for each delta value. Cases with identical scores were excluded. Orange segments indicate cases with discordance in the “peripheral vascular disease” category.

**Figure 5:**
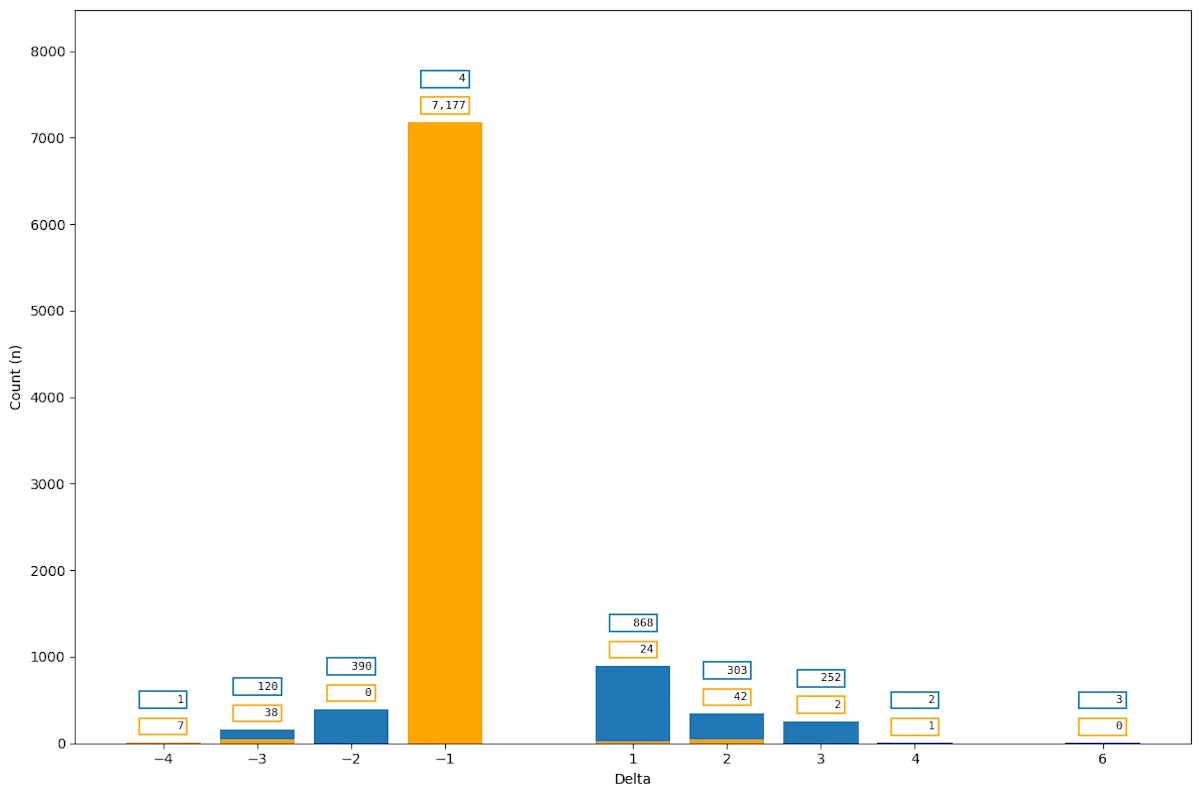
Distribution of score differences between the 2 mappings after filtering the data to only include the cases with a malignant disease diagnosis. Delta represents the score difference between our mapping and the mapping by Quan et al [12], calculated as score assigned by our mappings minus the score assigned by the mapping by Quan et al [12]. Bars show the number of cases for each delta value. Cases with identical scores were excluded. Orange segments indicate cases with discordance in the “peripheral vascular disease” category.

Figure 4 shows the distribution of score differences. The cases with identical scores (0 on the x-axis) were excluded for clarity. The orange segments of the bar chart show the cases with discordance in the “peripheral vascular disease” category. Because this category contributes 1 point to the CCI, it was most prominent among cases with this score differences and accounted for the overwhelming majority of these cases. Figure 5 shows the distribution after filtering the data to only include the observations with a malignant disease, showing how prominent a single category discordance can be in specific patient cohorts.

All cases in which the mapping by Quan et al [12] yielded a positive category score and ours did not can be explained by *ICD* codes that were intentionally excluded for inappropriateness. A detailed account of these codes is provided in Table 3. The most frequently observed code was Z95.81, accounting for 48.9% (7896/16,136) of all observed discordances. In the 2024 *ICD-10-GM*, Z95.81 denotes the presence of a surgically implanted vascular catheter system (eg, Broviac catheter or port system). Consequently, this code accounted for most discordances in the peripheral vascular disease category. Further examples—and very evident ones—would be the codes I85.0, I85.9, and I86.4 as the definition by the BfArM [21] states that these codes are to be used when the underlying condition is other than a liver disease.

**Table 3:** Discordant results between our mapping and that by Quan et al [12]^a^.

Category and *ICD-10*^b^ code			Mapping under which it was scored		Frequency, n		Description
**Peripheral vascular disease**							
	Z95.81	Quan et al [12]		7896		Presence of a surgically implanted vascular catheter system (Broviac catheter or port system)	
	I73.9	Quan et al [12]		379		Peripheral vascular disease, unspecified; includes arterial spasm and intermittent claudication not otherwise specified excluding peripheral arterial occlusive disease	
	K55.82	Quan et al [12]		37		Angiodysplasia of the small intestine with bleeding	
	K55.81	Quan et al [12]		15		Angiodysplasia of the small intestine without mention of bleeding	
	K55.88	Quan et al [12]		12		Other vascular diseases of the intestine	
	Z95.80	Quan et al [12]		10		Presence of a cardiac assist system (artificial heart or extracorporeal, intracorporeal, or paracorporeal pump)	
	K55.8	Quan et al [12]		8		Other vascular diseases of the intestine	
**Mild liver disease**							
	K74.71	Quan et al [12]		446		Liver cirrhosis, Child-Pugh class B	
	K74.72	Quan et al [12]		212		Liver cirrhosis, Child-Pugh class C	
	K71.0	Our mapping		748		Toxic liver disease with cholestasis	
	K76.1	Our mapping		221		Chronic congestive hepatopathy	
	K71.6	Our mapping		89		Toxic liver disease with hepatitis, not elsewhere classified	
	K71.9	Our mapping		66		Toxic liver disease, not elsewhere classified	
	K71.2	Our mapping		48		Toxic liver disease with acute hepatitis	
	K77.8	Our mapping		37		Liver diseases in other diseases classified elsewhere	
	K77.0	Our mapping		35		Liver diseases in infectious and parasitic diseases classified elsewhere	
	K77.22	Our mapping		9		Liver involvement in chronic graft-versus-host disease, stadium 2	
	K77.13	Our mapping		8		Liver involvement in acute graft-versus-host disease, stadium 3	
	K71.8	Our mapping		7		Toxic liver disease with other liver disorders	
	K77.23	Our mapping		5		Liver involvement in chronic graft-versus-host disease, stadium 3	
	K77.12	Our mapping		4		Liver involvement in acute graft-versus-host disease, stadium 2	
	K77.14	Our mapping		1		Liver involvement in acute graft-versus-host disease, stadium 4	
	K77.11	Our mapping		1		Liver involvement in acute graft-versus-host disease, stadium 1	
**Severe liver disease**							
	I85.9	Quan et al [12]		730		Esophageal varices without bleeding (excluding in liver diseases)	
	I85.0	Quan et al [12]		98		Esophageal varices with bleeding (excluding in liver diseases)	
	I86.4	Quan et al [12]		58		Gastric varices (excluding in liver diseases)	
	I98.2	Quan et al [12]		27		Esophageal and gastric varices without mention of bleeding (not stand-alone code)	
	K72.0	Our mapping		905		Acute and subacute hepatic failure, not elsewhere classified	
	K74.71	Our mapping		446		Liver cirrhosis, Child-Pugh class B	
	K74.72	Our mapping		212		Liver cirrhosis, Child-Pugh class C	
	K72.72	Our mapping		141		Hepatic encephalopathy grade 2	
	K72.73	Our mapping		111		Hepatic encephalopathy grade 3	
	K72.71	Our mapping		102		Hepatic encephalopathy grade 1	
	K72.79	Our mapping		56		Hepatic encephalopathy, grade unspecified	
	K72.74	Our mapping		26		Hepatic encephalopathy grade 4	
**Paraplegia and hemiplegia**							
	G83.2	Quan et al [12]		997		Monoparesis and monoplegia of an upper extremity	
	G83.1	Quan et al [12]		734		Monoparesis and monoplegia of a lower extremity	
	G83.41	Quan et al [12]		63		Incomplete cauda equina syndrome	
	G83.49	Quan et al [12]		47		Cauda equina syndrome, unspecified	
	G83.3	Quan et al [12]		28		Monoparesis and monoplegia, unspecified	
	G83.40	Quan et al [12]		16		Complete cauda equina syndrome	
	G80.0	Our mapping		78		Spastic quadriplegic cerebral palsy	
	G83.5	Our mapping		4		Locked-in syndrome	
	G83.80	Our mapping		1		Other specific paralytic syndromes	

^a^Presented are all the discordant International Classification of Diseases (ICD) codes from the first 4 categories (as shown in Table 2 and Figure 2). The rest were not presented here for clarity of the material. The complete version of the table can be found in Multimedia Appendix 1. Code descriptions are based on the original Federal Institute for Drugs and Medical Devices ICD, 10th Revision, German Modification, definitions (translated from German by the authors).

^b^*ICD-10: International Classification of Diseases, 10th Revision*.

## Discussion

### Principal Results

We created year-specific *ICD-10-GM* mappings (2004-2026) via dual independent review with consensus and implemented them in an open-source Python library. The new mappings not only included corrected alignment of codes to the comorbidity categories but also introduced Boolean rules that allowed for an even more precise algorithm.

Applied to 515,827 inpatient cases from the years 2010 to 2024, the new mappings showed excellent agreement with the mapping by Quan et al [12] (ICC=0.992; *P*<.001), whereas 3.1% (16,136/515,827) of cases received different total CCI scores.

Discordance was highly concentrated in a single administrative code—Z95.81 (presence of an implanted port system)—which was present in almost half (7896/16,136, 48.9%) of all discordant cases; because this code is nondiagnostic for peripheral vascular disease, it was excluded in our mappings.

Other codes were excluded because their BfArM [21] definitions clearly indicated that they were not diagnostic of the respective comorbidity category.

By applying those changes, and after inspecting individual discordant codes, we conclude that our new, year-specific mappings more precisely reflect the coding reality in the German health care system.

### Discordant Codes

We find it important to discuss some of the contents of Table 3 and the exclusion of selected codes that, in our opinion, are not diagnostic for the categories.

The most abundant discordant code, Z95.81, stands for the presence of a surgically implanted vascular catheter system (eg, Broviac catheter or port system). As the presence of a vascular access device does not, by itself, indicate peripheral vascular disease, this finding underscores the necessity of adapting automated comorbidity scoring tools to reflect contemporary coding definitions.

Codes starting with I85 denote esophageal varices. For codes I85.0, I85.9, and I86.4, the code definitions by BfArM [22] state clearly that they are not to be used if the underlying condition is liver disease. The coding guidelines designate combination codes I98.2 and I98.3 for these conditions, which were included in our definition via Boolean operators. In contrast, the *ICD-10, Clinical Modification* (*ICD-10-CM*), introduced codes for secondary esophageal varices as I85.10 and I85.11 in 2007 [23]. The definition by Quan et al [12] would include primary esophageal varices that are not due to underlying liver diseases both in current German and US coding standards.

Code I73.9 is described as “peripheral vascular disease” and was not scored as such in our mapping. This is because the code does not include claudication as a consequence of occlusion disease but, rather, describes functional causes such as arterial spasm [24].

Codes K74.71 and K74.72 were not scored in the category “mild liver disease” using our mapping but in “severe liver disease” as they describe advanced stages of liver cirrhosis [25].

K55.8 and its subcodes were excluded because, according to the *ICD-10-GM* classification, ischemic intestinal vascular diseases are specifically represented by K55.0 (“acute vascular disorders of intestine”) and K55.1 (“chronic vascular disorders of intestine”) [26]. K55.8 is reserved for other specified vascular diseases of the intestine not captured by these ischemic categories and was not considered equivalent to intestinal ischemia for the purpose of this mapping.

Z95.80, the presence of a cardiac assist system, does not unambiguously imply the condition of the peripheral vessels, which is why this code was excluded from the “peripheral vascular disease” category. Similarly, the current version of the *ICD-10-CM* includes code Z95.810, which signifies the “presence of automatic (implantable) cardiac defibrillator” [27], which would also not be diagnostic for the comorbidity.

Cauda equina syndrome—codes G83.40, G83.41, and G83.49—does not inherently imply paraplegia or hemiplegia, which is why we removed it.

### Limitations

The version of the CCI that we chose was the modification by Deyo et al [10], not the original one presented by Charlson et al [3] in 1987. We decided to use this modification because of the already available *ICD-10* mappings and their modifications, such as the one by Quan et al [12]. However, the original index with 2 additional comorbidity categories could deliver better, more precise results. This would require further investigation. Our transparent tooling could readily accommodate this in future analyses.

It is important to note that even a near-perfect algorithm and mappings are still dependent on the quality of clinical documentation and *ICD* coding practices. A Canadian 20-year validation of *ICD-10* administrative data using the CCI found that several comorbidities were increasingly undercoded over time [28]. Mapping accuracy cannot offset systematic undercoding, but errors compound. Each stage—documentation, coding, and mapping—matters for the validity and quality of retrospective observational research.

Even though modern statistical methods, often supported by—but not limited to—machine learning algorithms, can yield very cohort-specific comorbidity indexes with excellent local calibration, this methodology sacrifices comparability across studies and settings. General indexes such as the CCI enable risk adjustment and cross-study synthesis in national systems outside Europe, as shown in the Korean claim-based validation and scoping review by Shin and Han [29]. We therefore view tailored models as complementary to, not replacements for, standardized indexes such as the CCI.

### Conclusions

We provide year-specific, versioned mappings that enable programmatic calculation of the CCI using German administrative database codes (*ICD-10-GM*). In a direct comparison with the widely used mapping by Quan et al [12], agreement was very high.

Inspection of the discordant codes suggests that the discrepancies reflect clinically implausible inclusions in the older mapping when applied to current *ICD-10-GM* definitions.

The obtained results demonstrate that country- and year-specific mappings are necessary to avoid misclassification when legacy mappings are applied to contemporary German coding practice and that a small set of high-frequency administrative codes can substantially bias automated scoring at scale. The released, versioned mappings and lightweight Python implementation enable reproducible, year-appropriate CCI scoring. Our work provides valuable practical tools for CCI scoring when using German administrative data.

The calculation of comorbidity indexes from *ICD* codes is not novel per se, with the first publications dating back to 1992 [10]. The novelty of our work lies in updating the mapping to reflect the country- and year-specific *ICD-10* codes used in the German health system and making it easily accessible, interoperable, reusable, and adaptable for future modifications.

This study quantifies, to our knowledge for the first time, the magnitude and origin of mapping-related misclassification risk when outdated mappings are applied to current coding practices—demonstrating that maintaining country- and year-specific mappings is critical for valid risk adjustment.

The mappings are designed to be regularly updated just as the *ICD-10* iterations are released every year. The implemented architecture, which stores the code lists in separate, open-format files, intentionally supports these updates. It also allows for the addition of new comorbidity scores (eg, that by Elixhauser or the original CCI).

Performance is bounded by and dependent on the coding quality of the processed data. The present work targeted the *ICD-10-GM* for the years 2004 to 2026, the implication being that it requires yearly maintenance and alignment to match the changes in coding practices as the German *ICD-10* list is updated every year.

For CCI scoring in German datasets, locale- and version-specific mappings should be preferred over international lists. The case of a single code being present in almost 50% of discordances (Z95.81) demonstrated that a single change may have significant implications for overall scoring when processing data programmatically. This impact might even be increased in specialized patient subcohorts.

When reporting comorbidity adjustment, the CCI variant and the applied mapping should be stated. In studies using *ICD*-coded data, implementations often follow the mapping by Deyo et al [10] via the *ICD* mapping by Quan et al [12], yet this is frequently left implicit and only becomes clear through the cited works. We therefore recommend clearly documenting (1) the CCI specification used (original 19-category CCI vs 17-category modification by Deyo et al [10]) and (2) the mapping source and its version to ensure reproducibility and comparability across studies, for example, “Comorbidities were assessed using the original 19-item Charlson Comorbidity Index; scoring was performed by manual chart review” or “Comorbidities were assessed using the 17-item Deyo modification of the Charlson Comorbidity Index; scoring was performed automatically from ICD-10-CM codes using Quan et al’s mapping from 2005.”

## Appendix

Multimedia Appendix 1 Complete list of discordant results between our mapping and the mapping by Quan et al [12]. Code descriptions are based on the original Federal Institute for Drugs and Medical Devices (Bundesinstitut für Arzneimittel und Medizinprodukte) *International Classification of Diseases, 10th Revision*, German Modification definitions (translated from German by the authors). The categories for which no discordant codes were found in the dataset were left out.

## Abbreviations

BfArM: Federal Institute for Drugs and Medical Devices (Bundesinstitut für Arzneimittel und Medizinprodukte)
CCI: Charlson Comorbidity Index
ICC: intraclass correlation coefficient
ICD: International Classification of Diseases
ICD-10: *International Classification of Diseases, 10th Revision*
ICD-10-CM: *International Classification of Diseases, 10th Revision*, Clinical Modification
ICD-10-GM: *International Classification of Diseases, 10th Revision*, German Modification
ICD-9-CM: *International Classification of Diseases, 9th Revision*, Clinical Modification

## Data Availability

The data analyzed in this study contain pseudonymized patient information from the University Medical Center Mannheim and are therefore not publicly available due to legal and ethical restrictions. Access to the data may be granted on reasonable request to the University Medical Center Mannheim subject to institutional review and approval and applicable data protection regulations. The mapping files and the source code for calculation are openly available under the MIT License [31].
