# Supplementary material for: Developing Country-Specific Charlson Comorbidity Index Mappings for Use With German Administrative Data: Methodological Comparative Study
Source: JMIR Med Inform. 2026 Sep 3;14:e93923. doi: 10.2196/93923 (PMC13586706; doi:10.2196/93923)
Supplement: Supplementary file 1 [file medinform_v14i1e93923_app1.docx]

| **Category** | **ICD-10 Code** | **Scored by (Our / Quan)** | **Frequency** | **Description** |
| --- | --- | --- | --- | --- |
| Peripheral vascular disease | Z95.81 | Quan | 7896 | Presence of a surgically implanted vascular catheter system (Broviac catheter, port system) |
|  | I73.9 | Quan | 379 | Peripheral vascular disease, unspecified; includes arterial spasm, claudicatio intermittens not otherwise specified.; excluding peripheral arterial occlusive disease |
|  | K55.82 | Quan | 37 | Angiodysplasia of the small intestine with bleeding |
|  | K55.81 | Quan | 15 | Angiodysplasia of the small intestine without mention of bleeding |
|  | K55.88 | Quan | 12 | Other vascular diseases of the intestine |
|  | Z95.80 | Quan | 10 | Presence of a cardiac assist system (artificial heart, extracorporeal/intracorporeal/paracorporeal pump) |
|  | K55.8 | Quan | 8 | Other vascular diseases of the intestine |
| Mild liver disease | K74.71 | Quan | 446 | Liver cirrhosis, Child-Pugh B |
|  | K74.72 | Quan | 212 | Liver cirrhosis, Child-Pugh C |
|  | K71.0 | Our | 748 | Toxic liver disease with cholestasis |
|  | K76.1 | Our | 221 | Chronic Congestive Hepatopathy |
|  | K71.6 | Our | 89 | Toxic liver disease with hepatitis, not elsewhere classified |
|  | K71.9 | Our | 66 | Toxic liver disease, not elsewhere classified |
|  | K71.2 | Our | 48 | Toxic liver disease with acute hepatitis |
|  | K77.8 | Our | 37 | Liver diseases in other diseases classified elsewhere |
|  | K77.0 | Our | 35 | Liver diseases in infectious and parasitic diseases classified elsewhere |
|  | K77.22 | Our | 9 | Liver involvement in chronic graft-versus-host disease, stadium 2 |
|  | K77.13 | Our | 8 | Liver involvement in acute graft-versus-host disease, stadium 3 |
|  | K71.8 | Our | 7 | Toxic liver disease with other liver disorders |
|  | K77.23 | Our | 5 | Liver involvement in chronic graft-versus-host disease, stadium 3 |
|  | K77.12 | Our | 4 | Liver involvement in acute graft-versus-host disease, stadium 2 |
|  | K77.14 | Our | 1 | Liver involvement in acute graft-versus-host disease, stadium 4 |
|  | K77.11 | Our | 1 | Liver involvement in acute graft-versus-host disease, stadium 1 |
| Severe liver disease | I85.9 | Quan | 730 | Esophageal varices without bleeding (excl. in liver diseases) |
|  | I85.0 | Quan | 98 | Esophageal varices with bleeding (excl. in liver diseases) |
|  | I86.4 | Quan | 58 | Gastric varices (excl. in liver diseases) |
|  | I98.2 | Quan | 27 | Esophageal and gastric varices without mention of bleeding (not standalone code) |
|  | K72.0 | Our | 905 | Acute and subacute hepatic failure, not elsewhere classified |
|  | K74.71 | Our | 446 | Liver cirrhosis, Child-Pugh B |
|  | K74.72 | Our | 212 | Liver cirrhosis, Child-Pugh C |
|  | K72.72 | Our | 141 | Hepatic encephalopathy grade 2 |
|  | K72.73 | Our | 111 | Hepatic encephalopathy grade 3 |
|  | K72.71 | Our | 102 | Hepatic encephalopathy grade 1 |
|  | K72.79 | Our | 56 | Hepatic encephalopathy, grade unspecified |
|  | K72.74 | Our | 26 | Hepatic encephalopathy grade 4 |
| Paraplegia and Hemiplegia | G83.2 | Quan | 997 | Monoparesis and monoplegia of an upper extremity |
|  | G83.1 | Quan | 734 | Monoparesis and monoplegia of a lower extremity |
|  | G83.41 | Quan | 63 | Incomplete cauda equina syndrome |
|  | G83.49 | Quan | 47 | Cauda equina syndrome, unspecified |
|  | G83.3 | Quan | 28 | Monoparesis and monoplegia, unspecified |
|  | G83.40 | Quan | 16 | Complete cauda equina syndrome |
|  | G80.0 | Our | 78 | Spastic quadriplegic cerebral palsy |
|  | G83.5 | Our | 4 | Locked-in syndrome |
|  | G83.80 | Our | 1 | Other specific paralytic syndromes |
| Chronic Pulmonary Disease | J84.1 | Our | 383 | Other interstitial pulmonary diseases with fibrosis |
|  | J96.10 | Our | 316 | Chronic respiratory failure, not elsewhere classified, hypoxemic |
|  | J96.11 | Our | 140 | Chronic respiratory failure, not elsewhere classified, hypercapnic |
|  | J96.19 | Our | 108 | Chronic respiratory failure, not elsewhere classified, unspecified |
|  | T86.81 | Our | 95 | Lung transplant |
|  | J96.1 | Our | 55 | Chronic respiratory failure, not elsewhere classified |
|  | J99.23 | Our | 47 | Stage 3 chronic lung graft-versus-host disease |
|  | J84.10 | Our | 28 | Other interstitial pulmonary diseases with fibrosis, acute exacerbation specified |
|  | J84.11 | Our | 4 | Other interstitial pulmonary diseases with fibrosis, no acute exacerbation specified |
|  | J99.22 | Our | 4 | Stage 2 chronic lung graft-versus-host disease |
|  | J99.21 | Our | 2 | Stage 1 chronic lung graft-versus-host disease |
| Heart Failure | I42.2 | Our | 762 | Other hypertrophic cardiomyopathy |
|  | I42.1 | Our | 265 | Hypertrophic obstructive cardiomyopathy |
|  | I42.3 | Our | 6 | Eosinophilic endomyocardial disease |
|  | I42.4 | Our | 3 | Endocardial fibroelastosis |
| Dementia | G31.0 | Our | 165 | Localized cerebral atrophy |
|  | U63.4 | Our | 5 | Agitation or aggression in dementia |
|  | U63.6 | Our | 2 | Wandering behavior in dementia |
|  | U63.0 | Our | 1 | Psychotic symptoms in dementia |
| Any Malignancy | C86.5 | Our | 50 | Angioimmunoblastic T-cell lymphoma |
|  | C86.0 | Our | 21 | Extranodal NK/T-cell lymphoma, nasal type |
|  | C86.6 | Our | 16 | Primary cutaneous CD30-positive T-cell proliferations |
|  | C86.4 | Our | 5 | Blastic NK-cell lymphoma |
|  | C86.2 | Our | 5 | Enteropathy-type T-cell lymphoma |
|  | C86.1 | Our | 3 | Hepatosplenic T-cell lymphoma |
| Renal Disease | I13.20 | Our | 16 | Hypertensive heart and kidney disease with congestive heart failure and renal failure, without mention of hypertensive crisis |
|  | Z49.0 | Quan | 13 | Preparation for dialysis |
|  | I13.21 | Our | 3 | Hypertensive heart and kidney disease with congestive heart failure and renal failure, with mention of hypertensive crisis |
| AIDS | U60.3 | Our | 20 | Presence of AIDS indicator diseases (AIDS-defining conditions) |
